# Multiple Cavernous Haemangioma of Orbit and Cranium: A Case Report

**DOI:** 10.31729/jnma.5606

**Published:** 2021-01-31

**Authors:** Purnima Rajkarnikar Sthapit, Gita Sayami, Rohit Saiju

**Affiliations:** 1 Department of Oculoplasty and Ocular Oncology, Tilganga Institute of Ophthalmology, Kathmandu, Nepal; 2 Department of Pathology, HAMS Hospital, Kathmandu, Nepal

**Keywords:** *cavernous haemangioma*, *cranial haemangioma*, *multiple haemangiomas*, *orbital haemangioma*

## Abstract

A 32-year-old male presented with painless proptosis and diminution of vision in left eye. Imaging shows multiple well-defined masses, suggestive of cavernous haemangioma, in orbit and cranium with adjoining bones being thickened with cystic spaces. Histopathology proved the diagnosis. Cavernous haemangioma usually presents as a solitary intraconal and sometimes extraconal mass with vision usually preserved unless it extends to the apical portion. Here we report a rare case of multiple simultaneous locations of cavernous haemangiomas in orbit and cranium with significant diminution of vision.

## INTRODUCTION

Cavernous hemangioma is the most typical orbital tumor in adults in their third or fourth decade. It usually presents as a solitary tumor rarely affecting the vision. Treatment is straightforward with complete excision by orbitotomy surgery. However, multiple cavernous hemangiomas of orbit and cranium are rare but possible presentations, making it a diagnostic dilemma and management difficulty.

## CASE REPORT

A 32-year-old male presented with left sided gradual onset painless progressive forward protrusion of eyeball associated with gradual onset diminution of vision in same eye since four years.

On examination, the best-corrected visual acuity in the right eye (RE) was 20/20, while in the left eye (LE) was 20/400. Anterior segment and fundus examination of RE was within normal limits.

**Figure 1:**
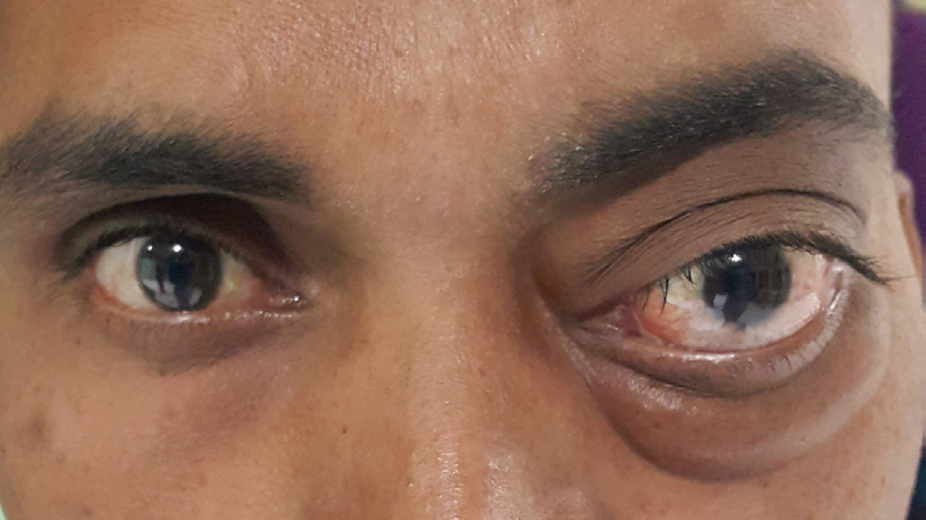
Patient presenting with proptosis and edema of eyelids and conjunctiva of left eye.

Examination of LE shows the fullness of the upper and lower lid with prominent eccentric proptosis. The eyeball was protruded four mm axial and three mm downward displaced, as shown in Figure 1. No increase of proptosis was noted on the Valsalva maneuver. Retropulsion of the globe was negative. However, extraocular movements were normal. LE also had a relative afferent pupillary defect and temporal disc pallor. The patient did not give any history of neurological illness like seizures.

**Figure 2:**
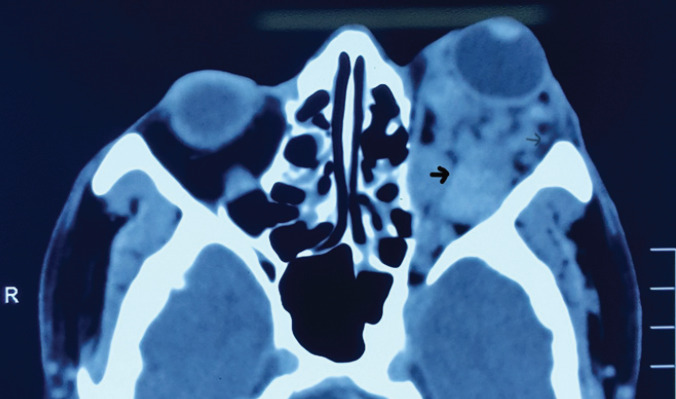
Plain CT scan orbit. Axial view showing multiple (at least 16) well defined round to oval, intra and extraconal masses in the left orbit-size ranged from 0.4 (small arrow) to 2.3 cm (big arrow) in largest diameter.

**Figure 3:**
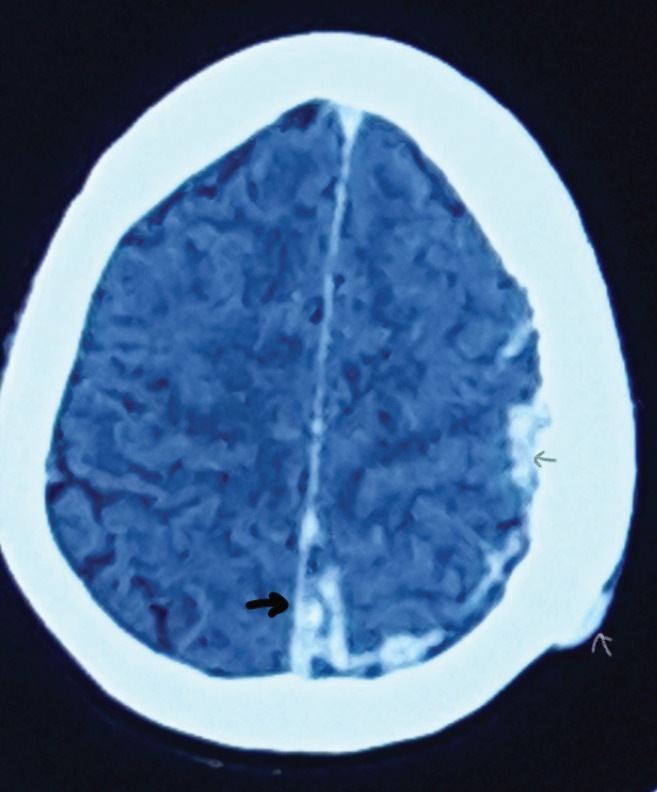
Plain CT scan brain axial view showing similar lesions in tentorium, falx (thick black arrow), dural surface (thin black arrow) and scalp (white arrow) of same side.

**Figure 4:**
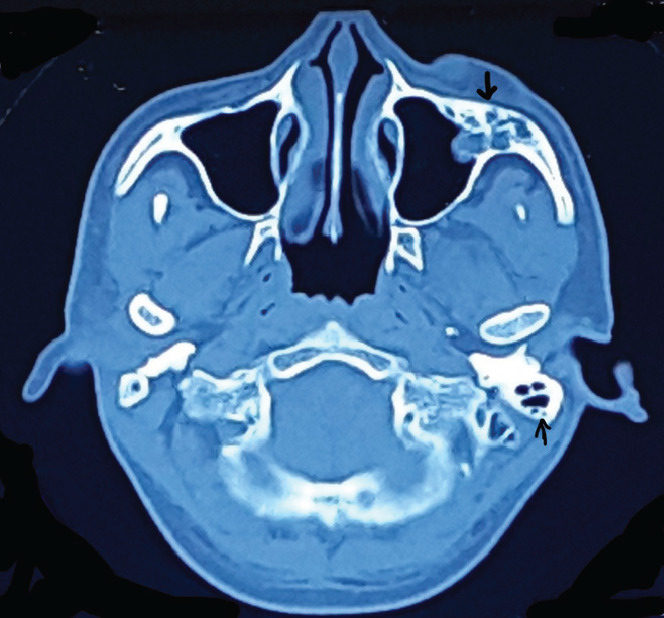
CT scan axial view bone window view showing thickened left parietal (thin arrow), maxillary and zygomatic bone (thick arrow) of left orbit with cystic spaces giving “honeycomb” appearance.

Figures 2,3 and 4 show CT scan brain and orbit with multiple (at least 16) well-defined round to oval, intra, and extraconal masses in the left orbit-size ranged from 0.4 to 2.3 cm in largest diameter. Similar lesions were seen in tentorium, falx cerebri, and scalp as well. The left zygomatic, frontal, and parietal bones were found to be thickened with cystic spaces. He was posted for lateral orbitotomy for excision biopsy of the lesions with the provisional diagnosis of multiple cavernous hemangiomas of orbit.

**Figure 5:**
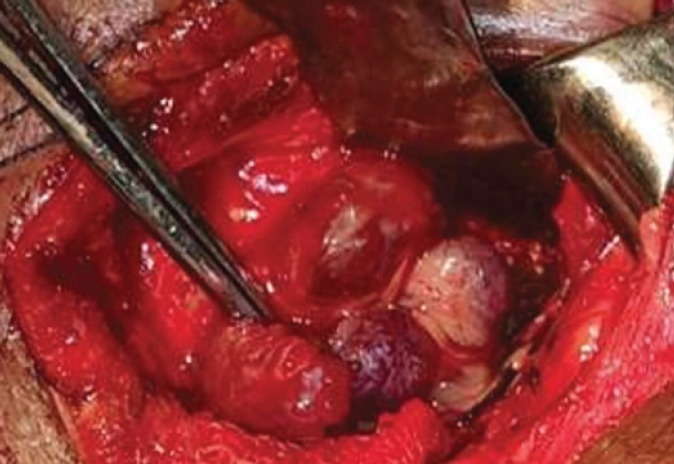
Intraoperative finding-multiple solid, solitary, dark red coloured lesions.

**Figure 6:**
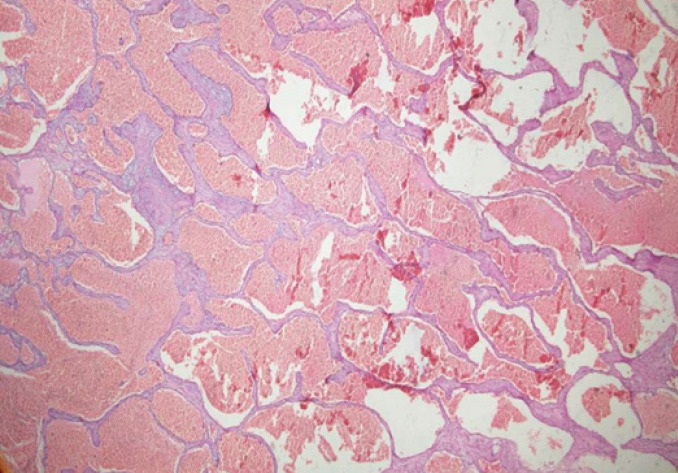
Hematoxylin and Eosin stain showing a thin walled vascular channels.

Intraoperatively, we found multiple solid, solitary dark red colored lesions (Figure 5), which were removed as much as possible. The histopathology slide in Figure 6 shows cystic thin-walled vascular channels lined by bland endothelial cells confirming Cavernous haemangioma's diagnosis. Vision in LE remained 20/400 postoperatively. Neurosurgical consultation confirmed the cranial lesions as the same. Given no neurological symptoms, they advised no intervention but to wait and watch.

## DISCUSSION

Cavernous hemangioma is the most common benign vascular tumor of the orbit in adults.^1^ Alan Mc Nab et al. states that it usually presents as a solitary intraconal lesion in 87% and sometimes as extraconal mass. In their study in 104 patients, all had single cavernous hemangioma in various parts of the orbit.^2^ A study also shows that 33% of patients have optic nerve dysfunction as well,^3^ which is likely in our case as well in view of reduced vision, RAPD, and disc pallor. Multiple cerebral cavernous hemangiomas have been reported in a child presenting with seizures, with most lesions located in supratentorial, frontal, and temporal lobes. These remain asymptomatic until intracranial hemorrhage occurs.^4^ Therefore, a close follow-up is required in a case like ours where intracranial hemorrhage may occur.

Multiple intraosseous cavernous haemangiomas similar to our patient is also reported by other studies in various locations like skull, spine, and inferior orbital rim.^5,6,7,8^ However, ours is the only case report with simultaneous presentation of multiple and multifocal presentations of cavernous haemangioma of orbit and cranium. According to a report by Brunereau L et al., our patient may have had the hereditary type of this disease given the tendency to form multiple lesions is seen in the hereditary rather than the sporadic type,^8^ however he had no family history. Complete surgical excision is a preferred treatment. Gamma knife radiotherapy and stereotactic radiation have also been tried for surgically challenging apical cases impeaching optic nerve with 76% reduction in the tumor volume.^9^ We would like to conclude by saying that cavernous haemangioma can rarely present multiple lesions in multiple sites in and outside the orbit.
